# DFN-kcr: a dual-branch deep learning model with attention-guided fusion for predicting lysine crotonylation sites in human non-histone proteins

**DOI:** 10.3389/fcell.2026.1877411

**Published:** 2026-07-29

**Authors:** Xin Wei, Siqin Hu, Chen Lin

**Affiliations:** 1 Business School, Jiangxi Institute of Fashion Technology, Nanchang, China; 2 School of Mega Data, Jiangxi Institute of Fashion Technology, Nanchang, China

**Keywords:** crotonylation sites, deep learning, human non-histone proteins, multi-head attention mechanism, residual transformer

## Abstract

**Introduction:**

Lysine crotonylation (Kcr) is extensively present in human non-histone proteins and plays a critical regulatory role in essential biological processes, including cell signaling and metabolic regulation. However, conventional wet-lab approaches for Kcr site identification are costly, time-consuming, and ill-suited for large-scale profiling. Although computational prediction methods have garnered increasing attention in recent years, there remains a notable lack of efficient and specialized tools tailored specifically for Kcr site prediction in human non-histone proteins.

**Methods:**

To address this gap, we propose DFN-Kcr, a dual-branch deep learning model explicitly designed for human non-histone Kcr site prediction. DFN-Kcr employs a dual-input strategy combining ProteinBERT embeddings and integer-encoded amino acid sequences, leveraging residual convolutional networks to capture local sequence motifs and a Transformer architecture to model long-range contextual dependencies. A branch-level gating attention fusion mechanism is further introduced to effectively integrate complementary features from both branches.

**Results:**

Extensive experiments demonstrate that DFN-Kcr significantly outperforms state-of-the-art methods, with its sensitivity (Sn), specificity (Sp), accuracy (Acc), and Matthews correlation coefficient (MCC) being 0.8244, 0.7679, 0.7961, and 0.5932, respectively.

**Discussion:**

This model offers a reliable solution for high-throughput identification of Kcr sites in non-histone proteins. To facilitate community access, we have deployed a user-friendly web server at http://www.lzzzlab.top/dfnkcr/.

## 1 Introduction

Lysine crotonylation (Kcr) is a recently identified post-translational modification that plays a critical role in cell signaling, metabolic regulation, gene expression, and cell cycle progression (Tan et al., 2011). Unlike the extensively studied acetylation, crotonylation involves a larger acyl group (the crotonyl group), which more substantially alters the charge and spatial configuration of the lysine residue, thereby exerting distinct effects on protein function (Westerveld et al., 2025). In recent years, crotonylation on histones has been demonstrated to be closely associated with spermatogenesis, tissue damage repair, and various diseases (e.g., cancer and neurodegenerative disorders) (Hou et al., 2021; Wan et al., 2019) However, the biological functions and regulatory mechanisms of crotonylation sites in human non-histone proteins remain to be deeply investigated (Xu et al., 2017). Accumulating evidence indicates that crotonylation of non-histone proteins also participates in the regulation of key metabolic enzyme activities and signal transduction pathways (Jiang et al., 2021; Wei et al., 2025); therefore, systematic identification of crotonylation sites in human non-histone proteins holds significant scientific importance.

Traditionally, the identification of Kcr sites has mainly relied on wet-lab techniques such as liquid chromatography-tandem mass spectrometry (LC-MS/MS) (Chen et al., 2025). Although these methods offer high accuracy, they are often costly, labor-intensive, time-consuming, and not suitable for large-scale high-throughput detection (Gao et al., 2024). Moreover, variations in experimental conditions may lead to false-positive or false-negative results (Jiang et al., 2024). Consequently, the development of efficient and reliable computational prediction methods has become a prominent focus in current bioinformatics research (Wang et al., 2026; Wei et al., 2017).

Prior to the widespread application of deep learning, the computational identification of Kcr sites primarily relied on classical machine learning frameworks. These methods typically encode protein sequences numerically and then employ classifiers to discriminate between positive and negative samples. In 2017 (Qiu et al., 2018), developed the iKcr-PseEns predictor, which incorporates coupling information into a pseudo-amino acid composition framework and integrates five independent random forest classifiers through a voting mechanism. In 2021 (Chen et al., 2021), provided a comprehensive review of six existing Kcr site prediction methods and, by integrating multiple feature encodings, constructed the CNNrgb deep learning framework specifically for identifying Kcr sites in human non-histone proteins. However, classical machine learning methods generally suffer from two inherent limitations: first, feature extraction is heavily dependent on manual design, making it difficult to capture intrinsic information within sequences; second, when the training sample size increases or the distribution of positive and negative samples becomes severely imbalanced, the generalization ability of the model declines significantly. These issues remain limitations of traditional machine learning to this day.

In recent years, with the advancement of deep neural networks in proteomics, many researchers have begun applying them to Kcr site prediction (Lv et al., 2021a). developed the Deep-Kcr method, which integrates sequence features, physicochemical properties, and numerical spatial information, combined with feature selection, achieving promising results on large-scale data. In 2022 (Qiao et al., 2022), proposed a model called BERT-Kcr, which uses a pre-trained BERT to convert amino acid sequences into context-aware embedding vectors, followed by a bidirectional LSTM for classification; the ten-fold cross-validation results demonstrated good performance. The same year (Khanal et al., 2022),developed the DeepCap-Kcr classifier, which replaced traditional CNNs with a capsule network to better capture the spatial hierarchical relationships among amino acid residues. In 2024 (Jiang et al., 2024), constructed a model named PlantNh-Kcr for crotonylation sites in plant non-histone proteins, combining CNN, BiLSTM, and attention mechanisms, and tested it on multiple plant datasets including wheat, tobacco, and rice. That same year (Zuo et al., 2024), proposed ILCROsite, which employs an MLP with stacked self-attention and introduces an under-sampling method to address the issue of imbalanced positive and negative samples. In 2025 (Wei et al., 2017), introduced iKcr-DRC, whose main architecture is a densely connected convolutional network for extracting high-level local features, coupled with an enhanced channel attention mechanism that imparts residual characteristics to the network. Also in that year (Wang et al., 2026), combined the pre-trained protein language model ESM2 with BiLSTM, using focal loss to handle imbalanced data, achieving high-accuracy prediction of crotonylation sites in plant non-histone proteins with interpretability, and outperforming existing methods. Looking back at the development of deep learning, from CNNs to RNNs, and then to Transformers and pre-trained language models, model capabilities have been steadily improving. However, how to make predictions more interpretable and how to adapt models to differences across species remain outstanding challenges (Gong and Qu, 2025; Li et al., 2026). Although current machine learning and deep learning methods have made some progress in Kcr site prediction, several issues persist: specialized tools for human non-histone proteins are largely lacking, and prediction performance still has room for improvement.

**FIGURE 1:**
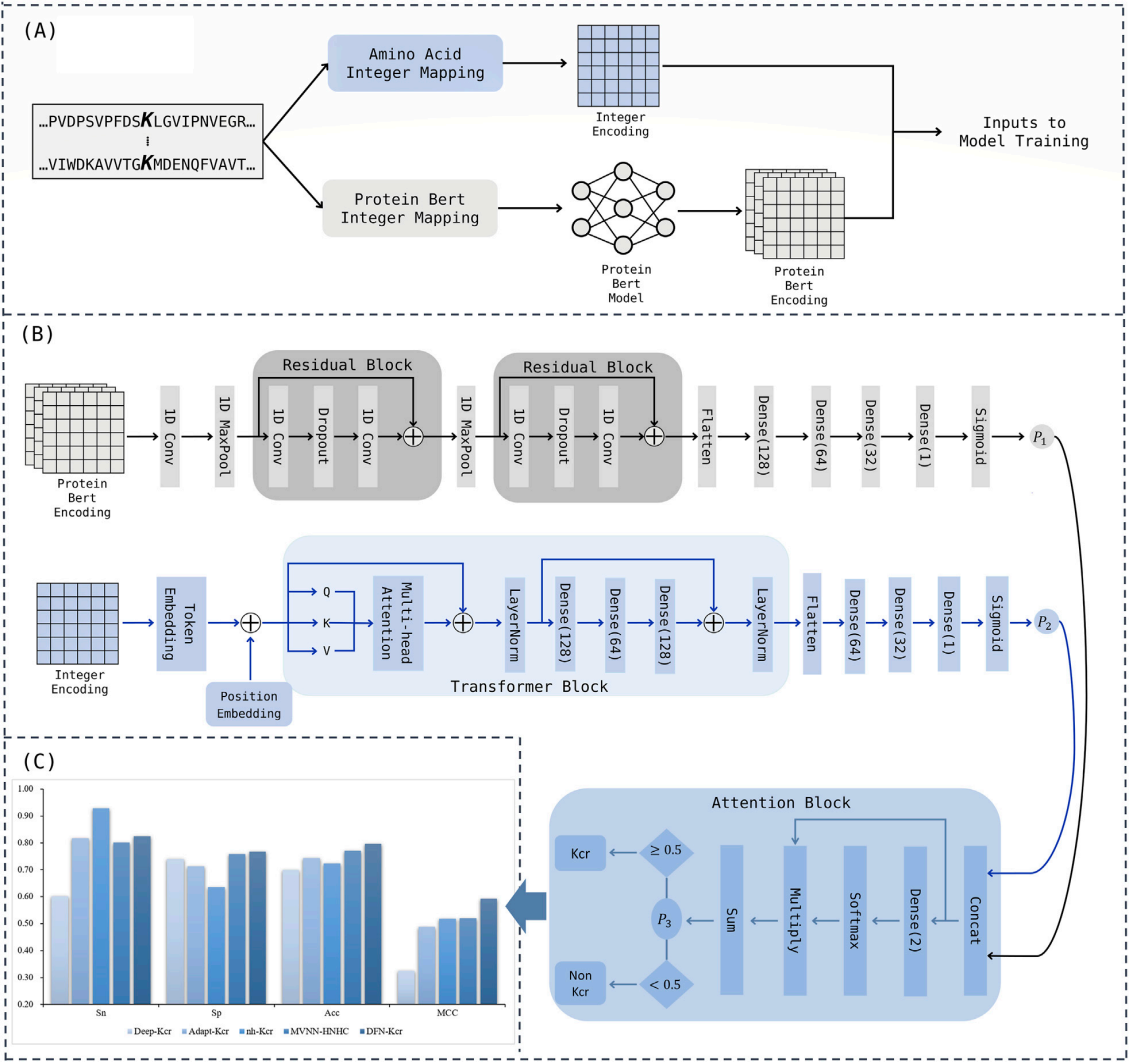
Framework of DFN-Kcr. **(A)** Dual-branch encoding strategy converting protein sequences into ProteinBERT embeddings and integer encodings. **(B)** Model architecture with CNN and Transformer branches integrated via branch-level gating attention fusion. **(C)** Performance comparison with existing predictors on the independent test set.

In response to the aforementioned bottlenecks and challenges, we focus on the prediction of Kcr sites in human non-histone proteins and propose a dual-branch fusion deep learning model, Dual-branch Fusion Network for Kcr Prediction (DFN-Kcr). This model overcomes the limitations of single-model approaches in feature extraction. Specifically, we design two input pathways: ProteinBERT and amino acid integer encoding. On one pathway, a residual convolutional network accurately captures local motif features from the sequence; on the other, a Transformer fully exploits long-range context dependencies. Together, these pathways enable more comprehensive and efficient extraction of protein sequence features. To address the potential performance inconsistency between the two branches when making predictions independently, we introduce an attention-guided feature fusion module that adaptively adjusts the weights of the two branches. This renders the model more stable and its results more objective when facing complex sample distributions. Compared with traditional machine learning and single-architecture deep learning methods, DFN-Kcr achieves significantly improved accuracy and robustness in Kcr site identification through complementary feature learning from the two branches coupled with adaptive fusion. This model not only provides a useful tool for precise prediction of post-translational modification sites but also offers a novel technical perspective for mechanistic studies of epigenetics-related modifications. Figure 1 illustrates the overall workflow of DFN-Kcr. The dataset and source code used in this study are available at https://github.com/weixin7112/dfn_kcr.

## 2 Materials and methods

### 2.1 Benchmark dataset

**FIGURE 2:**
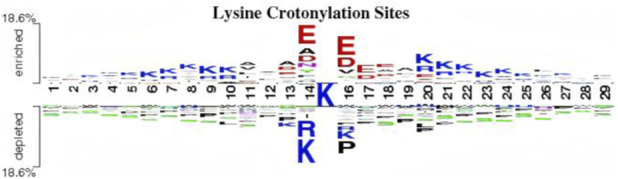
Two-sample logo showing statistically significant differences in positional residue preferences between modified and unmodified K sites in the dataset.

The original protein sequences and annotation information used in this study were obtained from the UniProt database. On this basis, we directly adopted the benchmark dataset of human non-histone crotonylation sites constructed and publicly released by (Gao et al., 2024). The positive samples in this dataset were all validated by mass spectrometry experiments. For negative samples were randomly selected randomly selected from the same group of human non-histone proteins, specifically lysine residues not annotated as crotonylation sites. These negative lysines were required to be located in sequence regions with similar local environments to the positive sites. To avoid potential bias, the number of negative samples was set to approximately five times that of positive samples, reflecting the natural imbalance of crotonylation events. To eliminate the interference of redundant sequences on model evaluation, Gao et al. used CD-HIT [22] to remove redundancy with a sequence similarity threshold of 30%. Each sequence sample was centered on the crotonylated lysine residue, with 14 residues extracted both upstream and downstream, resulting in a fixed-window fragment of 29 amino acid residues. For sequences shorter than the window length, a padding strategy using the unknown amino acid X was applied. The entire dataset was divided into a cross-validation set and an independent test set, with sample distributions detailed in Table 1. Furthermore, site-specific analysis of positive and negative samples was performed using the Two Samples Logo online tool, and the results are shown in Figure 2.

**TABLE 1:** Original sample data.

Original dataset	Positive	Negative
Training dataset	12,262	60,101
Testing dataset	3,343	15,010
Total	15,605	75,111

### 2.2 Feature extraction methods

#### 2.2.1 Amino acid integer encoding

Amino Acid Integer Encoding (AAIE) is a commonly used feature extraction method in bioinformatics, whose core idea is to map amino acid residues to consecutive integer codes. Specifically, for the 20 standard amino acid types, each amino acid is represented by a specific integer. For example, alanine (Ala, A) can be represented as 0, cysteine (Cys, C) as 1, and so on. In this study, each peptide segment centered on the crotonylated lysine residue has a length of 29 residues. After applying Amino Acid Integer Encoding, each sample is converted into a 29 × 1 vector. The integer-encoded sequence is then passed through an embedding layer that maps each integer to a learnable dense vector before being input to the residual CNN. The advantage of this encoding method lies in its ease of implementation, no requirement for prior knowledge, and its ability to preserve the categorical information of amino acids, thereby providing a raw sequential representation for the subsequent embedding layer.

#### 2.2.2 ProteinBert encoding

ProteinBert Encoding (PBE) is a deep language model specifically designed for protein sequences, proposed by (Brandes et al., 2022). Its pre-training scheme innovatively combines two tasks: Masked Language Model (Devlin et al., 2019) and Gene Ontology (Ashburner et al., 2000; The Gene Ontology, 2026) annotation prediction, enabling the model to capture sequence patterns while also sensing protein functional label information. At the architectural level, ProteinBERT introduces a novel design that separates local and global representations, allowing it to handle inputs and outputs of varying scales in a unified end-to-end manner; thus, it achieves both efficiency and flexibility when processing long sequences. Owing to these design choices, despite its model size being substantially smaller than that of contemporary deep learning methods, ProteinBERT attains performance close to or even surpassing existing methods on multiple benchmark tasks, including protein structure prediction, post-translational modification recognition, and biophysical property prediction.

In this study, we employ the ProteinBERT pre-trained model for sequence feature extraction. First, the 20 standard amino acids, selenocysteine (U), other amino acids, and the padding character “X” are respectively mapped to integer codes in the range of 0–22. Subsequently, based on the pre-trained weights provided by the official open-source code repository (https://github.com/nadavbra/protein.bert), we construct the PB-EM (ProteinBert-Embedding Module) sub-module. This sub-module consists of an input layer stacked with an embedding layer: the input layer receives the integer-encoded peptide sequence and performs length normalization, while the embedding layer converts the normalized discrete tokens into dense vectors of dimension 128. After inputting a peptide sequence of length 29 into the PB-EM module, the model outputs a protein embedding feature matrix of shape 29 × 128, denoted as PBE, which serves as the input feature for the subsequent deep network.

For the two encoding schemes, we adopted different processing pathways. On the amino acid integer encoding side, each peptide segment of length 29 is directly converted into a 29 × 1 vector, which serves to retain the categorical information of the amino acids. For ProteinBERT encoding, we leverage the pre-trained embedding module to output a 29 × 128 dense feature matrix, which captures the contextual meaning of the sequence. The features obtained from the two pathways are fed into the network in parallel, ultimately combining local and global information in a complementary manner.

### 2.3 Model construction

Considering the substantial difference in data morphology between the amino acid integer encoding and ProteinBERT encoding, we designed a dual-branch heterogeneous network, equipping each type of input with a dedicated decoding and feature extraction architecture.

#### 2.3.1 Residual convolutional network-based protein sequence feature extraction module

This module is responsible for extracting deep local features from the embedding vectors generated by the ProteinBERT pre-trained model, corresponding to the gray branch in Figure 1B. The input tensor first passes through a 1D convolutional layer for initial feature mapping, followed by a 1D max-pooling layer to reduce the sequence length, which both decreases computational cost and retains key features.

The core processing part consists of two stacked residual blocks (He et al., 2016). The internal structure of each residual block is as follows:

1. Two consecutive convolution-normalization-convolution sub-layers, each sub-layer sequentially comprising a 1D convolution (filters = 64, kernel_size = 3, strides = 1, padding = ‘same’), Dropout, and a 1D convolution.
2. Residual connection: the input signal is directly added via a “shortcut” to the output of the last convolutional layer. Assuming the input is $x_{i}$ and the final convolutional mapping is $F\left(x_{l}\right)$ , then the output is obtained as shown in Equation 1:

$$H\left(x_{l}\right)=F\left(x_{l}\right)+x_{i}$$ (1)

After passing through the two residual blocks, a 1D max-pooling layer is further applied to compress the feature map. Finally, the high-dimensional features are flattened and fed into a four-layer fully connected network (with the number of neurons decreasing layer by layer) for further feature extraction, outputting the probability $P_{1}$ .

#### 2.3.2 Sequence feature extraction module based on a self-attention mechanism

Transformer (Vaswani et al., 2017) is a deep learning architecture driven by the self-attention mechanism, specifically designed to address long-range dependency problems in sequence modeling. Unlike traditional recurrent neural networks, Transformers do not rely on recursive structures and can efficiently extract global contextual information through parallel computation. Owing to their powerful representational capacity, Transformers have achieved considerable success in numerous fields, including natural language processing, computer vision, and bioinformatics (Consens et al., 2025; Dosovitskiy et al., 2020; Floridi and Chiriatti, 2020).

In this study, we employed a Transformer-based encoding architecture to extract features from integer encoding-encoded protein sequences, corresponding to the blue branch in Figure 1C. The specific computational steps are as follows.

1. Input embedding and positional embedding encoding. To map the amino acid integer encoding into a continuous latent space while preserving the sequential position information, this branch employs two layers of embedding: token embedding and positional embedding.
  Token embedding: A linear projection layer is used to convert the amino acid integer encoding into a dense embedding $Z_{\text{token}}\in R^{L\times d_{\text{model}}}$ , where L is the sequence length and $d_{\text{model}}$ is the token embedding dimension.
  Positional embedding: To enable the model to perceive the order of amino acids in the sequence, learnable positional embeddings $E_{\text{pos}}\in R^{L\times d_{\text{model}}}$ are introduced.
  Finally, the token embedding and the positional embedding are added element‑wise to obtain the final input, as shown in Equation 2:
$$Z_{0}=E_{\text{token}}+E_{\text{pos}}$$ (2)
2. The core of the Transformer block is the multi-head self-attention mechanism, which captures long-range dependencies and contextual relationships among amino acid residues across the entire sequence. For an input Z , the $Q_{h}$ , $K_{h}$ , and $V_{h}$ matrices are first computed for each attention head $h\in \left\{1,\ldots ,H\right\}$ according to Equation 3.
$$\left\{\begin{array}{c} Q_{h}=Z_{0}\cdot W_{h}^{Q}\\ K_{h}=Z_{0}\cdot W_{h}^{K}\\ V_{h}=Z_{0}\cdot W_{h}^{V} \end{array}\right.$$ (3)
  Here, $W_{h}^{Q}$ , $W_{h}^{K}$ , and $W_{h}^{V}\in R^{L\times d_{k}}$ are learnable weight matrices, and $d_{k}=d_{\text{model}}/H$ . Subsequently, the computation for the *hth* attention head is performed as shown in Equation 4.
$$A\text{ttention}\left(Q_{h},K_{h},V_{h}\right)=\text{SoftMax}\left(\frac{Q_{h}{K_{h}}^{T}}{\sqrt{d_{k}}}\right)V_{h}$$ (4)
  The outputs from the H attention heads are concatenated and then learned (i.e., linearly transformed) to obtain the final attention output, as shown in Equation 5.
$$\text{MHSA}\left(Z_{0}\right)=\text{concat}\left(A\text{ttention}\left(Q_{1},K_{1},V_{1}\right),\ldots ,A\text{ttention}\left(Q_{H},K_{H},V_{H}\right)\right)\cdot W_{\text{MHSA}}$$ (5)
  where $W_{\text{MHSA}}\in R^{d_{\text{model}}\times d_{\text{model}}}$ is a learnable weight matrix, and $\text{concat}\left(\cdot \right)$ denotes the tensor concatenation operation. After applying the residual connection (adding the input to the attention output) and then layer normalization (LayerNorm), the output is obtained as shown in Equation 6:
$$Z_{1}=\text{LayerNorm}\left(Z_{0}+\text{MHSA}\left(Z_{0}\right)\right)$$ (6)
  Subsequently, $Z_{1}$ is fed into a feed-forward layer (FFL) for further feature transformation. This network consists of three fully connected layers with 128, 64, and 128 neurons, respectively. Finally, after again applying a skip connection (residual connection) and layer normalization, the final output of the final output of the Transformer block is obtained as shown in Equation 7:
$$Z_{2}=\text{LayerNorm}\left(Z_{1}+\text{FFL}\left(Z_{1}\right)\right)$$ (7)
3. The output $Z_{2}$ of the Transformer block is passed through a series of fully connected layers to map the high-dimensional sequence features to a probability output $P_{2}$ .

#### 2.3.3 Dual-branch attention fusion module

To fully integrate the complementary predictive information from the CNN branch $P_{1}$ and the Transformer branch $P_{2}$ , this study designed a branch-level gating attention fusion module that achieves efficient integration of the two branches’ prediction results through adaptive weight assignment, ultimately outputting the final prediction probability $P_{3}$ for Kcr sites. The attention mechanism here operates on branch-level output vectors rather than token-level sequence representations, serving as a lightweight gating function to weight the contribution of each branch. The specific computational procedures of this module are as follows:

1. Concatenation. The prediction outputs from the two independent branches are concatenated in terms of dimensions to construct a fused feature vector. Given that the output of the CNN branch is $P_{1}$ and the output of the Transformer branch is $P_{2}$ , the concatenation operation is defined in Equation 8.
$$P_{\text{concat}}={\left[P_{1},P_{2}\right]}^{T}$$ (8)
2. Attention weight calculation. The concatenated feature vector $P_{\text{concat}}$ is fed into a fully connected layer with a hyperbolic tangent activation function for nonlinear transformation. The transformed features are then mapped to normalized attention weights α via the SoftMax function, achieving adaptive importance assignment to the prediction results of the two branches. The entire computational process is shown in Equation 9.
$$\alpha =\text{SoftMax}\left(W_{1}\cdot P_{\text{concat}}\right)={\left[\alpha_{1},\alpha_{2}\right]}^{T}$$ (9)
  where $W_{1}$ is a learnable weight matrix.
3. Weighted fusion. The attention weights are element-wise multiplied with the original branch prediction results and then summed, completing the weighted feature fusion and outputting the final prediction probability $P_{3}$ as shown in Equation 10:
$$P_{3}=\alpha_{1}P_{1}+\alpha_{2}P_{2}$$ (10)
4. Based on the final prediction probability $P_{3}$ , the classification prediction is completed using Equation 11.

$$\text{Prediction}=\left\{\begin{array}{l} \text{Kcr},P_{3}\geq 0.5\;\\ \text{Non}-\text{Kcr},P_{3}<0.5 \end{array}\right.$$ (11)

### 2.4 Performance evaluation

For the binary classification task of Kcr site prediction, this study adopted four evaluation metrics: sensitivity (Sn), specificity (Sp), accuracy (Acc), and Matthews correlation coefficient (MCC) (Jia et al., 2023), whose definitions are shown in Equation 12.

$$\left\{\begin{array}{l} \text{ Sp}=\frac{\text{TN}}{\text{TN}+\text{FP}}\\ \text{ Sn}=\frac{\text{TP}}{\text{TP}+\text{FN}}\\ \text{ Acc}=\frac{\text{TP}+\text{TN}}{\text{TP}+\text{TN}+\text{FP}+\text{FN}}\\ \text{ MCC}=\frac{\text{TP}\times \text{TN}-\text{FP}\times \text{FN}}{\sqrt{\left(\text{TP}+\text{FP}\right)\times \left(\text{TP}+\text{FN}\right)\times \left(\text{TN}+\text{FP}\right)\times \left(\text{TN}+\text{FN}\right)}} \end{array}\right.$$ (12)

where True Positives (TP) is the number of samples that are actual Kcr sites and are also predicted as Kcr sites by the model; True Negatives (TN) is the number of samples that are actual non-Kcr sites and are also predicted as non-Kcr sites; False Positives (FP) is the number of samples that are actual non-Kcr sites but are predicted as Kcr sites by the model; False Negatives (FN) is the number of samples that are actual Kcr sites but are predicted as non-Kcr sites.

### 2.5 Training details

All algorithm implementations and comparative experiments in this study were carried out in a unified Python programming environment. The proposed dual-branch deep learning model, DFN-Kcr, was constructed and trained using the TensorFlow 2.0 framework, with hardware acceleration provided by an NVIDIA Tesla P100 GPU (16 GB memory) to improve the efficiency of model training and inference. The key settings, including network architecture parameters, training hyperparameters, and optimizer configurations, were determined through extensive tuning and comparison. The specific values and detailed settings are listed in Table 2. For further details, please refer to the source code at https://github.com/weixin7112/dfn_kcr.

**TABLE 2:** The key hyperparameter settings.

Hyperparameter	Value
$d_{model}$	128
H	2
Optimizer	AdamW
Epochs	150
Loss function	Binary cross entropy loss

## 3 Results and discussion

### 3.1 Performance comparison with different branch network architectures

On the basis of validating the independent representation learning capability of each branch, and to further clarify the performance advantages of the DFN-Kcr dual-branch fusion architecture over single-branch structures for the Kcr site prediction task, we designed a series of comparative experiments to systematically evaluate the overall performance of different branch network architectures, focusing on the impact of branch structure differences on predictive performance. To ensure fairness in the experimental comparisons, all baseline models were trained using the same training strategy as DFN-Kcr and evaluated using ten-fold cross-validation. The results are shown in Table 3.

**TABLE 3:** Performance comparison of different branch network architectures on 10-fold cross-validation.

Predictor	Sn	Sp	Acc	MCC	Precision
AAIE + Transformer	0.8348	0.7132	0.7740	0.5526	0.7447
PBE + CNN	**0.8474**	0.7306	0.7890	0.5828	0.7593
DFN-kcr	0.8442	**0.7430**	**0.7936**	**0.5908**	**0.7670**

The highest score for each metric is shown in bold.

As shown in Table 3, the DFN-Kcr dual-branch fusion model significantly outperforms both single-branch models across all evaluation metrics, fully demonstrating the effectiveness and superiority of the dual-branch synergistic fusion. Specifically, in terms of sensitivity, the PBE + CNN branch achieved the best performance (0.8474), followed by the AAIE + Transformer branch (0.8348), while DFN-Kcr reached 0.8442—slightly lower than that of PBE + CNN but still remaining at a high level, indicating its ability to efficiently capture positive class features. Regarding specificity, DFN-Kcr achieved 0.7430, which represents improvements of 4.1% and 1.7% over AAIE + Transformer (0.7132) and PBE + CNN (0.7306), respectively, demonstrating that the dual-branch fusion effectively enhances the model’s ability to discriminate and exclude non-Kcr sites. Furthermore, DFN-Kcr ranked first in the three comprehensive metrics: accuracy, MCC, and precision.

In summary, although the two single-branch models already exhibited good predictive performance, through the complementary integration of the dual-branch architecture, DFN-Kcr achieves synergistic utilization of multiple features, thereby further improving overall performance, strongly supporting the rationality and effectiveness of this fusion strategy.

### 3.2 Improvement of model performance by the attention fusion mechanism

To evaluate the impact of different feature fusion strategies on model performance, this study designed comparative experiments using three typical fusion mechanisms—arithmetic average fusion, maximum fusion, and attention fusion—while keeping the dual-branch network structure unchanged. To further assess the expressive capacity of the fusion module, two additional gate variations were tested: an MLP gate (2 → 8 → 2 with SoftMax) and a logits-based gate. The experiments were also conducted using ten-fold cross-validation, and the detailed performance comparison results are shown in Table 4.

**TABLE 4:** 10-fold cross-validation results of different fusion mechanisms.

Strategy	Sn	Sp	Acc	MCC	Precision
Average	0.8486	0.7288	0.7887	0.5818	0.7580
Max	**0.9011**	0.6535	0.7772	0.5727	0.7225
Attention (MLP)	0.8434	0.7404	0.7919	0.5873	0.7649
Attention (logits)	0.7894	**0.7823**	0.7858	0.5722	**0.7894**
Attention	0.8442	0.7430	**0.7936**	**0.5908**	0.7670

The highest score for each metric is shown in bold.

From the experimental results, all three fusion methods can integrate the predictive information from the dual-branch network for Kcr site recognition, but notable differences exist among them. Arithmetic average fusion and maximum fusion are both static methods, showing certain biases. Maximum fusion achieved the highest sensitivity (0.9011), indicating a tendency to classify samples as positive (Kcr sites), but its specificity was only 0.6535, reflecting weaker discriminative ability for negative samples and a higher risk of false positives, thereby limiting overall performance. Arithmetic average fusion yielded relatively more balanced metrics across all indicators.

In contrast, the attention fusion mechanism outperformed the other two static fusion methods across all evaluation metrics, demonstrating significantly better comprehensive performance. Specifically, attention fusion achieved a specificity of 0.7430, which is approximately 1.9% higher than arithmetic average fusion and 13.7% higher than maximum fusion, indicating a substantially enhanced ability to exclude non-Kcr negative sites. Its accuracy and precision were 0.7936 and 0.7670, respectively, representing improvements of 0.6% and 1.2% over arithmetic average fusion, and 2.1% and 6.2% over maximum fusion, thereby effectively improving overall prediction accuracy. Most notably, attention fusion achieved a MCC of 0.5908, the highest among the three strategies, demonstrating a better balance between positive class recognition and negative class exclusion.

Among the two attention variants, the MLP gate (2 → 8 → 2 with SoftMax) yielded a MCC of 0.5873, which are comparable to but slightly lower than those of the original attention gate. The logits-based gate, while improving specificity to 0.7823 and precision to 0.7894, suffered a notable drop in sensitivity and MCC, suggesting that operating directly on raw logits makes the gate overly conservative toward negative samples. Neither variant surpassed the original attention design in terms of overall performance.

These results fully demonstrate that the attention fusion mechanism can dynamically assign weights to the dual-branch network, adjusting the contribution of each branch according to the characteristics of different samples, thereby achieving better integration of features from both branches. In contrast, arithmetic average and maximum fusion rely on fixed rules and cannot adapt to sample variability, making it difficult to fully exploit the complementary advantages of the dual-branch network.

### 3.3 Comparison with classical machine learning methods and protein language models

To validate the advantages of the proposed architecture, we selected four classical machine learning algorithms (SVM, RF, LR, and DT) and three scales of fine-tuned ESM-2 (35M, 150M, and 650 M parameters) as baseline models. All classical machine learning models were implemented using the scikit-learn toolkit, trained with exactly the same input features as DFN-Kcr: the 2-D ProteinBERT embedding is first flattened into a 1-D vector and then concatenated with the integer encoding vector to form a unified feature representation. All models used default parameters. For ESM-2, a downstream classification head with a hidden layer of 256 units followed by a single output neuron was appended to each variant for binary classification. All experiments were also conducted using ten-fold cross-validation for performance evaluation, and the detailed results are shown in Tables 4, 5.

**TABLE 5:** Comparison of DFN-Kcr with traditional machine learning methods on 10-fold cross-validation.

Predictor	Sn	Sp	Acc	MCC	Precision
SVM	0.6671	0.4824	0.5747	0.1545	0.5635
RF	0.6906	0.6805	0.6855	0.3712	0.6838
LR	0.7048	0.6801	0.6924	0.3851	0.6878
DT	0.5846	0.6037	0.5941	0.1883	0.5961
DFN-kcr	**0.8442**	**0.7430**	**0.7936**	**0.5908**	**0.7670**

The highest score for each metric is shown in bold.

The DFN-Kcr model significantly outperformed all four classical machine learning baseline models across every evaluation metric, fully demonstrating the performance advantages of deep learning architectures for the Kcr site prediction task and further validating the rationality and effectiveness of the dual-branch fusion design of the DFN-Kcr model (Table 5). The primary reason for the limited performance of classical machine learning algorithms lies in their insufficient capacity to process high-dimensional input features, making them unable to effectively handle discrete encoding schemes such as amino acid integer encoding, and thus they cannot fully exploit the key biological information embedded in protein sequences. Furthermore, DFN-Kcr also substantially outperformed all fine-tuned ESM-2 variants, with the most notable improvement in MCC (Table 6). Although ESM-2_150 M achieved a marginally higher precision, our dual-branch architecture consistently delivered superior overall performance. These results indicate that fine-tuning a general-purpose protein language model alone is insufficient for Kcr site prediction and is surpassed by a task-specific design. In contrast, the DFN-Kcr model, through its dual-branch fusion architecture, fully combines the respective advantages of amino acid integer encoding and ProteinBERT encoding. The CNN branch efficiently captures local motif features of protein sequences, while the Transformer branch deeply mines long-range context dependencies. The attention fusion mechanism then achieves optimal integration of the two branches. Consequently, DFN-Kcr comprehensively surpasses classical machine learning algorithms across all performance metrics, further validating the superiority of deep learning architectures for the Kcr site prediction task and highlighting the practical value of the DFN-Kcr model.

**TABLE 6:** Comparison of DFN-Kcr with fine-tuned protein language models on 10-fold cross-validation.

Predictor	Sn	Sp	Acc	MCC	Precision
ESM2_35 M	0.7932	0.6503	0.7211	0.4502	0.6958
ESM2_150 M	0.7936	0.6501	0.7217	0.4492	**0.7896**
ESM2_650 M	0.7764	0.6696	0.7233	0.4502	0.7029
DFN-kcr	**0.8442**	**0.7430**	**0.7936**	**0.5908**	0.7670

The highest score for each metric is shown in bold.

### 3.4 Validation of representation learning capability of different branch network architectures

**FIGURE 3:**
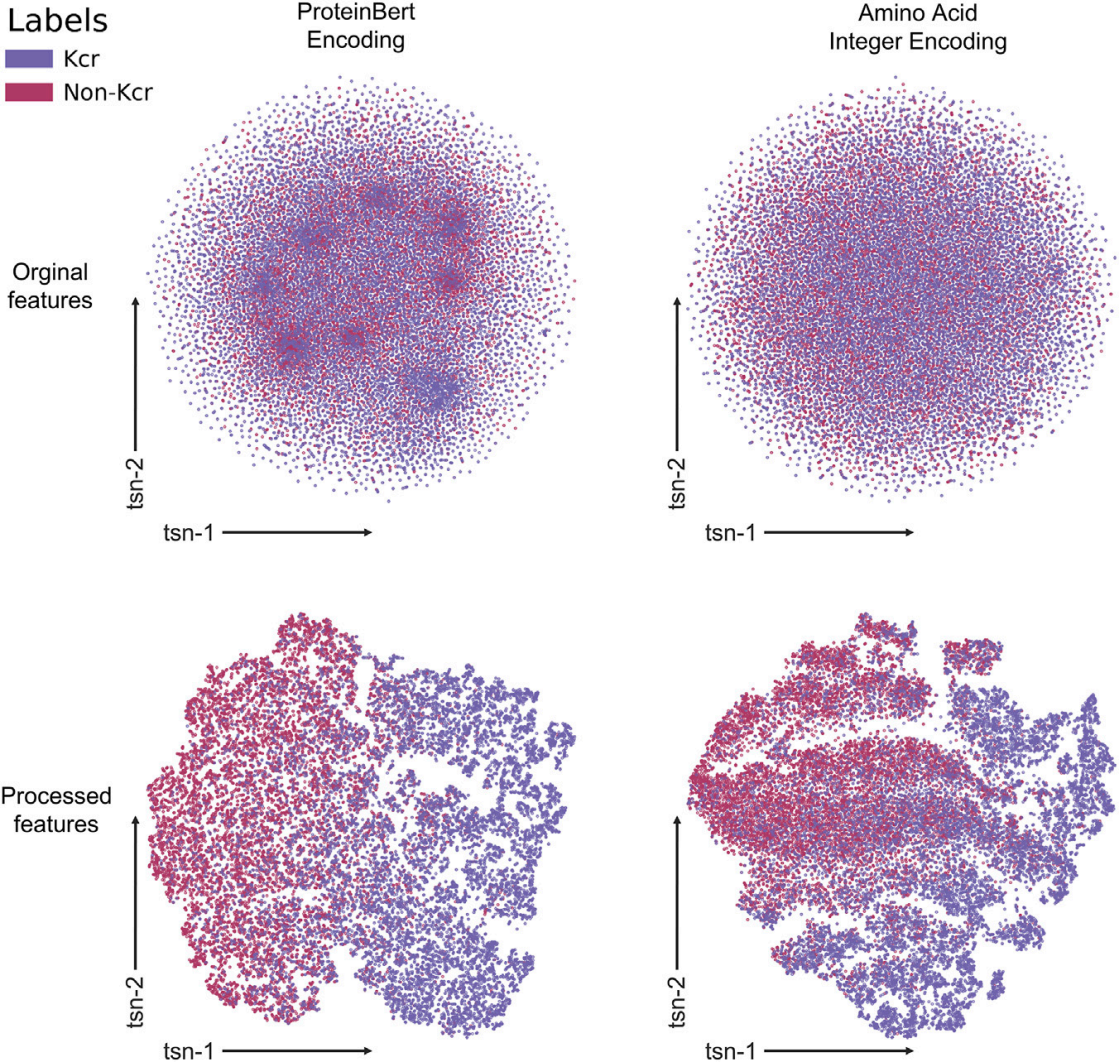
Comparison of t-SNE visualizations between raw features and deep features extracted by branch networks. Upper panels show raw features of ProteinBert encoding and amino acid integer encoding, with Kcr and non-Kcr sites heavily intermingled. Lower panels show deep features from the CNN branch and Transformer branch, with clear separation between the two classes.

To intuitively evaluate the effectiveness of the dual-branch network architecture in the DFN-Kcr model for feature representation learning, this study employed t-SNE dimensionality reduction and visualization. The original input features (including amino acid integer encoding and ProteinBERT encoding) and the deep features extracted by the corresponding branch networks were projected into two-dimensional space. The results are shown in Figure 3. In the figure, blue points represent Kcr positive samples, and red points represent non-Kcr negative samples; t-SNE-1 and t-SNE-2 denote the two coordinate dimensions after dimensionality reduction.

From the visualization of the original features, it can be seen that for both amino acid integer encoding and ProteinBERT encoding, the corresponding Kcr and non-Kcr samples exhibit a highly overlapping distribution in the two-dimensional space, with blurred class boundaries and severe mixing, making them almost non-separable. This observation indicates that the raw encoding features, without network modeling, are insufficient to effectively capture the intrinsic differences between Kcr sites and non-Kcr sites; if used directly for classification tasks, a model would struggle to learn discriminative feature representations. This also reveals, from the feature perspective, one of the fundamental reasons for the limited performance of classical machine learning methods in the Kcr site prediction task.

In contrast, the visualization results of the deep features extracted by the corresponding branch networks show that Kcr and non-Kcr samples exhibit a significant clustering separation trend in the two-dimensional space. For the deep features extracted by the ProteinBERT + CNN branch, the distribution regions of Kcr samples and non-Kcr samples are clearly distinguished. Similarly, the deep features extracted by the amino acid integer encoding + Transformer branch also demonstrate good separability, with substantially reduced mixing between the two classes, and samples from different classes are aggregated in distinct spatial regions.

These results fully validate the effectiveness of the two branch network architectures in the DFN-Kcr model: both the ProteinBERT + CNN branch and the amino acid integer encoding + Transformer branch possess strong feature representation learning capabilities, enabling them to extract highly discriminative deep features from the raw encoding features.

### 3.5 Comparison with existing kcr site prediction models

To further validate the state-of-the-art performance of the DFN-Kcr model in the Kcr site prediction task, five mainstream Kcr site prediction models published in recent years in the bioinformatics field were selected as baselines, including Deep-Kcr (Lv et al., 2021b), Adapt-Kcr (Li et al., 2022), nh-Kcr (Chen et al., 2021), and MVNN-HNHC (Gao et al., 2024). The experimental results of all models tested on the same dataset are shown in Table 7.

**TABLE 7:** Comparison of DFN-Kcr with existing predictors.

Predictor	Sn	Sp	Acc	MCC	Precision
Deep-kcr	0.6013	0.7402	0.6986	0.3257	—
Adapt-kcr	0.8173	0.7120	0.7436	0.4879	—
Nh-kcr	**0.9286**	0.6351	0.7232	0.5179	—
MVNN-HNHC	0.8006	0.7577	0.7706	0.5203	—
DFN-kcr	0.8244	**0.7679**	**0.7961**	**0.5932**	0.7796

The highest score for each metric is shown in bold.

From the comparison results, the DFN-Kcr model significantly outperforms all existing baseline models in overall performance, particularly excelling in the MCC metric, fully demonstrating its state-of-the-art performance in the Kcr site prediction task. Specifically, the MCC value of the DFN-Kcr model reaches 0.5932, which represents improvements of 26.75%, 10.53%, 7.53%, and 7.29% compared to Deep-Kcr, Adapt-Kcr, nh-Kcr, and MVNN-HNHC (the four baseline models), respectively, indicating substantially better classification stability and reliability than existing models.

In terms of sensitivity, the nh-Kcr model achieves the highest value, but its specificity is only 0.6351, showing a clear performance bias—overemphasizing positive site recognition at the expense of negative site exclusion, thereby limiting its overall performance. The sensitivity value of DFN-Kcr is 0.8244, which, although lower than that of nh-Kcr, remains at a high level while also maintaining strong negative class recognition. In terms of specificity, the DFN-Kcr model reaches 0.7679, the highest among all baseline models, indicating superior ability to exclude non-Kcr negative sites and better classification accuracy.

In terms of accuracy, the DFN-Kcr model achieves 0.7961, significantly higher than all baseline models, with relative improvements of 9.75%, 5.25%, 7.29%, and 2.55% over the existing models, respectively, intuitively demonstrating its advantage in overall predictive capability.

Comprehensive analysis reveals that existing models generally suffer from either performance bias or insufficient overall capability: some models focus heavily on positive class recognition, resulting in weak negative class exclusion; others attempt to balance both but achieve limited overall performance, making it difficult to achieve accurate and stable Kcr site prediction. In contrast, the DFN-Kcr model, through its dual-branch fusion architecture and attention fusion mechanism, effectively balances positive class recognition and negative class exclusion, thus achieving comprehensive improvements across all evaluation metrics and reaching the state-of-the-art level among existing Kcr site prediction models.

### 3.6 Performance on S/T phosphorylation site prediction

Furthermore, to further validate the generality of the DFN-Kcr architecture, we applied it to the serine/threonine (S/T) phosphorylation site prediction task using the dataset from (Lv et al., 2021b). The benchmark dataset contained 5,387 positive samples and 5,387 negative samples, which were randomly divided into a training set and an independent test set at a ratio of 8:2. A new phosphorylation-specific model was trained on this dataset using the DFN-Kcr architecture. The independent test results are shown in Table 8, where this model significantly outperformed the other three prediction models in terms of Sn, Acc, MCC, and AUC. Specifically, the model achieved improvements of 2.97% and 1.8% in the most important metrics, Sn and MCC, respectively. This suggests that the DFN-Kcr architecture possesses transferability across PTM types.

**TABLE 8:** Comparison of the serine/threonine (S/T) phosphorylation site dataset with other models.

Predictor	Sn	Sp	Acc	MCC	Precision
DeepPSP	0.7665	**0.8378**	0.8021	0.606	—
Bert-ST	0.8007	0.7460	0.7984	0.600	—
DeepIPs	0.7961	0.8350	0.8063	0.632	—
DFN-kcr	**0.8258**	0.8239	**0.8248**	**0.650**	0.8242

The highest score for each metric is shown in bold.

### 3.7 Web server for prediction

**FIGURE 4:**
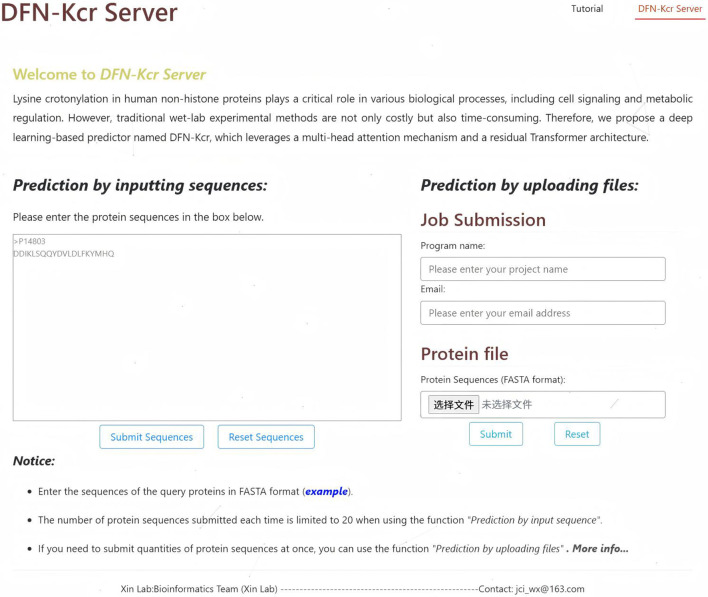
Server homepage.

For the convenience of research and use by experts and scholars, this study has established a web server. The homepage is shown in Figure 4. The following is a guide to using the server.

The protein sequence to be queried can be submitted in two ways. In the first approach, the user can directly enter the protein sequence in the center of the homepage and click the “Submit Sequences” button to obtain the prediction results. Alternatively, the user can upload a FASTA-formatted file using the option in the lower-right corner, specify a job name and the recipient’s email address in the same section, and then click the “Submit” button. The results will subsequently be sent to the provided email address.

## 4 Conclusion

In this study, we developed a dual-branch deep learning model, DFN-Kcr, for the prediction of Kcr sites in human non-histone proteins. The model adopts two input pathways: amino acid integer encoding and ProteinBERT encoding. Local sequence motifs are extracted via a residual convolutional network, while global context dependencies are captured using a Transformer architecture. An attention-guided fusion mechanism is introduced to adaptively integrate features from the two branches, effectively overcoming the limitations of single-network structures and static fusion methods in feature learning.

Experimental results demonstrate that DFN-Kcr significantly outperforms classical machine learning algorithms and existing mainstream Kcr prediction models across key metrics, including sensitivity, specificity, accuracy, precision, and MCC, achieving more balanced and stable predictive performance. t-SNE visualization further confirms that the deep features learned by the dual-branch network exhibit clear class separability, effectively distinguishing Kcr sites from non-Kcr sites. The attention fusion mechanism dynamically assigns branch weights, fully leveraging the complementary advantages of the two branches and avoiding the performance bias introduced by static methods such as average or maximum fusion. Importantly, the DFN-Kcr architecture yields competitive results on the S/T phosphorylation site prediction task after retraining on a specific phosphorylation site dataset, demonstrating its robust transferability across different PTM categories. This suggests that the architecture has strong potential for application to other PTM prediction tasks.

Although DFN-Kcr achieves promising predictive performance, there remains room for improvement. Currently, the model relies solely on sequence encoding information. Future work could integrate multi-source biological information, such as protein physicochemical properties, evolutionary conservation, and secondary structure, to construct a more comprehensive feature representation system. Moreover, the present model is focused on human non-histone proteins; subsequent efforts could extend to multi-species datasets to build a more general cross-species prediction framework.

In summary, DFN-Kcr enables more accurate prediction of Kcr sites in human non-histone proteins, providing a reliable computational tool for related bioinformatics research. The online web server established in this study supports researchers in conducting high-throughput predictions conveniently, offering effective support for subsequent experimental screening and functional validation.

## Data Availability

The data presented in the study are deposited in the https://github.com/weixin7112/dfn_kcr.
